# Broca's Region: Novel Organizational Principles and Multiple Receptor Mapping

**DOI:** 10.1371/journal.pbio.1000489

**Published:** 2010-09-21

**Authors:** Katrin Amunts, Marianne Lenzen, Angela D. Friederici, Axel Schleicher, Patricia Morosan, Nicola Palomero-Gallagher, Karl Zilles

**Affiliations:** 1 Research Centre Jülich, Institute for Neuroscience and Medicine (INM-1, INM-2), Jülich, Germany; 2 Jülich-Aachen Research Alliance (JARA), RWTH Aachen University, Department of Psychiatry and Psychotherapy, Aachen, Germany; 3 Max Planck Institute for Human Cognitive and Brain Sciences, Leipzig, Germany; 4 C. u. O. Vogt-Institute for Brain Research, University of Duesseldorf, Duesseldorf, Germany; New York University, United States of America

## Abstract

There is a considerable contrast between the various functions assigned to Broca's region and its relatively simple subdivision into two cytoarchitectonic areas (44 and 45). Since the regional distribution of transmitter receptors in the cerebral cortex has been proven a powerful indicator of functional diversity, the subdivision of Broca's region was analyzed here using a multireceptor approach. The distribution patterns of six receptor types using in vitro receptor autoradiography revealed previously unknown areas: a ventral precentral transitional cortex *6r1*, dorsal and ventral areas *44d* and *44v*, anterior and posterior areas *45a* and *45p*, and areas *op8* and *op9* in the frontal operculum. A significant lateralization of receptors was demonstrated with respect to the cholinergic M_2_ receptor, particularly in area *44v+d*. We propose a new concept of the anterior language region, which elucidates the relation between premotor cortex, prefrontal cortex, and Broca's region. It offers human brain homologues to the recently described subdivision of area 45, and the segregation of the ventral premotor cortex in macaque brains. The results provide a novel structural basis of the organization of language regions in the brain.

## Abstract 2

A novel map of Broca's language region is proposed based on transmitter receptor distributions as functionally relevant molecular markers. It sheds new light on the relation between anatomy and functional segregation.

## Author Summary

Broca's region is involved in many aspects of language processing in the brain. Such detailed functional diversity, however, is in contrast to its classical anatomical subdivision into only two cortical areas. Since the regional distribution of neurotransmitter receptors has been proven to be a powerful indicator of functional segregation, we revised the subdivision of Broca's region by analyzing the distribution of six different receptor types in the human brain. On the basis of these results, we propose a novel map of Broca's and neighboring regions with several, previously unknown areas. Moreover, a significant left-sided interhemispheric asymmetry of receptors was found, mainly for the cholinergic muscarinic M_2_ type. This asymmetry correlates with the well-known left-sided dominance for language. Finally, we present a model of the molecular organization of the anterior human language region and neighboring prefrontal and motor areas on the basis of similarities in their receptor patterns. This model contributes to our understanding of the relation between motor areas and classical Broca region. Our results are important for future studies of the functional segregation and the role of mirror neurons in the human brain, and are relevant for revealing homologies between human and macaque brains.

## Introduction

For more than a century, Broca's region in the posterior part of the inferior frontal gyrus has been considered essential for speech production [1]. Effortful, telegraphic speech, impairment in articulation and melodic line, semantic and phonemic paraphasias are some of the symptoms associated with lesions of this region and subsequent Broca's aphasia [2],[3]. Mohr et al. [4], however, showed that an infarction limited to Broca's region does not cause chronic speech production deficits, and thus, differs from the clinical characteristics in Broca aphasia. They concluded that Broca's aphasia is observed after damage that extends beyond Broca's region. Broca's pioneering study illustrates on the one hand the power of the clinico-anatomical approach, i.e., relating language functions to a brain region, but also demonstrates its limitations. Consequently, the anatomical correlates of Broca's region cannot be identified by lesion studies alone.

**Figure 1:**
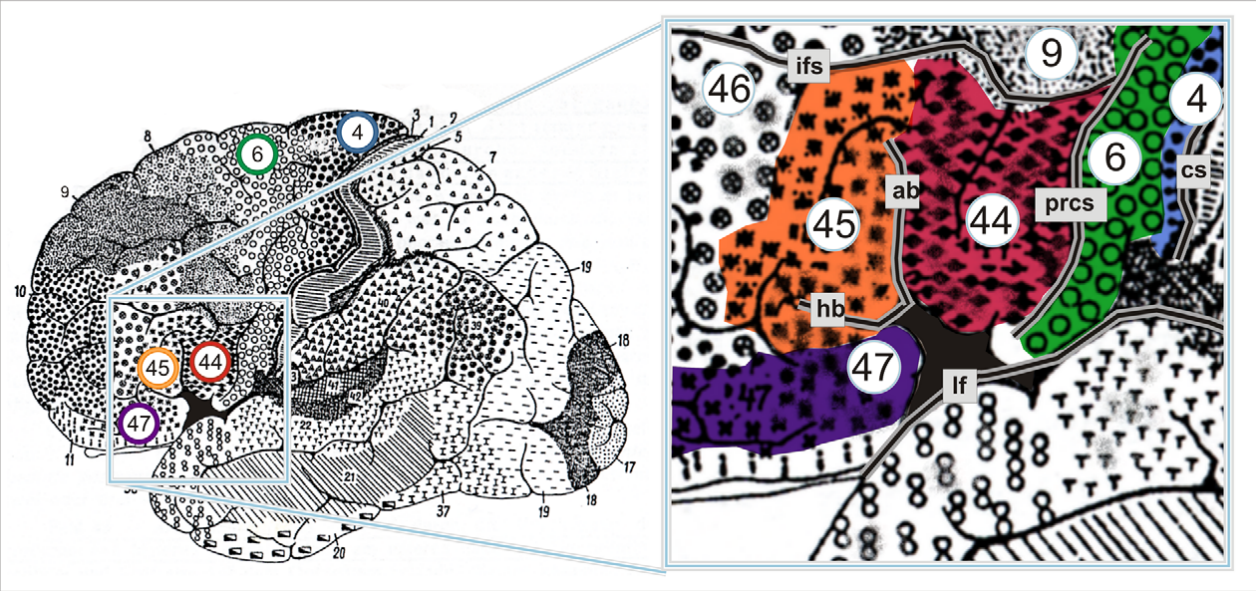
Cytoarchitectonic map of the lateral surface of a human cortex adapted from Brodmann [**5**]. The region of interest contains areas 44 and 45 as well as parts of the neighboring areas 4, 6, and 47. Note that Brodmann's map does not show the ventral border of area 44, 45, and 6 in the depth of the lateral fissure. ab, ascending branch of the lateral fissure; cs, central sulcus; hb, horizontal branch of the lateral fissure; ifs, inferior frontal sulcus; lf, lateral fissure; prcs, precentral sulcus.

According to Brodmann's map [5], the posterior part of the inferior frontal gyrus represents Broca's speech region. Brodmann's areas 44 and 45 at the opercular and triangular parts of the inferior frontal gyrus are its putative cytoarchitectonic correlates [6],[7]. Neighboring areas include premotor area 6 at the ventral precentral gyrus, dorso-lateral prefrontal areas 9 and 46, area 47 at the orbital part of the inferior frontal gyrus, and the anterior insula (Figure 1). Brodmann's map became a widely distributed anatomical reference for the interpretation of functional imaging studies although it represents only a schematic 2-D sketch of a putative “typical” human brain; i.e., it considers neither intersubject variability in brain anatomy nor interhemispheric asymmetries.

In contrast to the rather simple parcellation of the inferior frontal lobe shown in Brodmann's map, recent functional imaging studies suggest a complex segregation of Broca's region and neighboring areas of the inferior frontal cortex [8]–[16]. The whole region is involved in various aspects of language including phonological and semantic processing, action execution and observation, as well as music execution and listening (for an overview see e.g., [17]–[20]). A meta-analysis suggested that the opercular part (area 44) is particularly involved in syntactic processing [21]. However, activation during processing of syntactically complex sentences was also assigned to area 45 (triangular part) in studies using semantic plausibility judgment tasks or sentence picture-matching tasks [22],[23]. Other studies showed activation in area 44 in production [10] and comprehension [11],[12]. A recent study crossing the factors of semantics and syntax demonstrated that area 44 and more anterior areas (45/47) were active during sentence comprehension; area 44 carried the main effect of syntactic complexity independent of semantic aspects, whereas semantic relatedness, as well as its interaction with syntax, was located more anteriorly [24]. In addition, the deep frontal operculum was shown to be segregated from the inferior frontal gyrus during processing of syntactic sequences [25]. Finally, activations during motor tasks were also observed near Broca's region, e.g., during imagery of a motion task [14].

In many cases, the Brodmann map does not enable a localization of functional clusters of activations, in particular when they are found buried in the sulci, where architectonic borders have not been mapped. The localization of activation clusters using 3-D probabilistic cytoarchitectonic maps of areas 44 and 45 [26], and the adjoining motor areas [27], demonstrated that some of the clusters did not only overlap with area 44, but with the neighboring Brodmann area 6 [14].

A frequent finding in neuroimaging is a functional activation spot covering the adjoining border regions of two or more Brodmann areas, which cannot be assigned unequivocally to a cytoarchitectonic area. This situation may be caused by methodical problems of generating functional activation maps (e.g., spatial normalization to a template, smoothing, mislocalization of the BOLD signal due to venous flow) or by biological reasons (e.g., intersubject variability). Beside these arguments, it must also be asked whether Brodmann's map adequately represents the cytoarchitectonic segregation of this region, or whether uncharted cortical areas lead to the observed mismatch between functional data and cytoarchitecture as provided by Brodmann's map.

This line of argument is further supported by architectonic studies in the macaque brain. Recently, a new map of the ventral motor-prefrontal transitional region of the macaque cortex has been proposed; it showed that area F5 consists of three subareas: F5c, F5p, and F5a [28],[29]. Area F5 plays a major role in the mirror neuron system and has been interpreted as a putative correlate of human area 44 [30], whereas other authors disagreed [31],[32]. The complex segregation of the macaque ventral frontal cortex (and area F5 in particular) as compared to the rather simple subdivision of the human cortex provides further arguments to question Brodmann's parcellation.

Quantitative receptor autoradiography, a method that demonstrates the inhomogeneous regional and laminar distribution patterns of neurotransmitter receptor binding sites in the brain [33]–[35] has been proven to be a powerful mapping tool [34],[36]–[38]. The quantitative analysis of the density of multiple receptors in each cortical area highlights the regionally specific balance between different receptor types, and the differences between cortical areas. It reveals a functionally relevant parcellation, since receptors play a crucial role in neurotransmission [34].

Our aim was, therefore, to establish a receptor-based architectonic parcellation of the posterior inferior frontal cortex with focus on Broca's region, its right hemispheric homologue, and the adjoining areas on the frontal operculum, as well as the ventral premotor cortex. We studied the distribution patterns of six different receptor binding sites of four neurotransmitter systems: glutamatergic AMPA and kainate receptors, GABAergic GABA_A_ receptors, cholinergic muscarinic M_1_ and M_2_ receptors, and noradrenergic α_1_ receptors in autoradiographs of eight human brains (Table 1). Neighboring sections were stained for cell bodies in order to identify the cytoarchitecture in this region. Observer-independent receptor and cytoarchitectonic mapping methods [34] combined with multivariate statistics were applied to analyze the similarity and dissimilarity of receptor patterns between the cortical areas. As a result, three previously unknown areas and a further segregation of the classical Broca areas 44 and 45 were found. The study leads to a new organizational concept of the cortical areas in Broca's region. It demonstrates that motor cortex, Broca's region, and prefrontal areas differ in their regionally specific receptor expression patterns, and thus in their signal processing properties.

**Table 1:** Summary of the post mortem brains.

Protocol Number	Hemisphere	Orientation of the Section	Age (y)	Sex	Cause of Death	Post Mortem Delay (h)	Brain Weight (g)^a^
MR3	r	Coronal	79	m	Sudden cardiac death, chronic cardiac insufficiency	12	1,326
MR2	l	Coronal	75	f	Bronchial cancer	16	1,280
MR1	l	Coronal	78	m	Multiorganic failure	12	1,477
HG 02/01	l/r	Coronal	77	f	Pulmonary edema	18	1,128
HG 05/00	r	Coronal	72	m	Cardiac arrest	8	1,326
HG 02/98	l/r	Horizontal	63	f	Suffocation	23	1,172
HG 03/97	l/r	Horizontal	56	m	Cardiac arrest	15	1,340
HG 24/96	l/r	Horizontal	80	f	Cardiac arrest	10	1,100

a Fresh weight.

f, female; m, male; r, l, left and right hemispheres.

## Results

**Figure 2:**
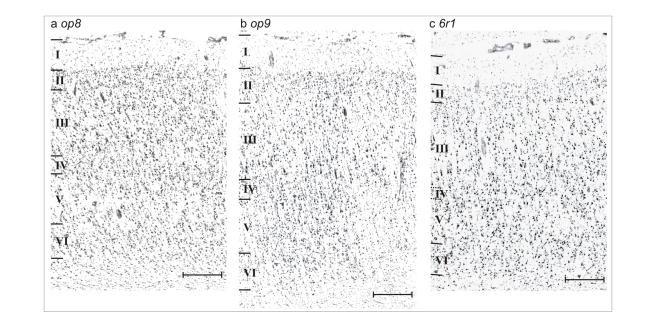
Photomicrographs of cell body–stained cryostat sections of areas *op8* (a), *op9* (b), and *6r1* (c). The frontal opercular areas *op8* and *op9* are both dysgranular (i.e., thin lamina IV). The cell packing density was slightly larger in area *op8* than in area *op9*. Area *6r1* has an even thinner layer IV; it is almost agranular. Its laminar pattern is weak. In contrast to ventral area 6 and Broca's area 44 it contains smaller pyramidal cells in lamina III. Scale bar, 0.5 mm. Roman numerals indicate the cortical layers.

**Figure 3:**
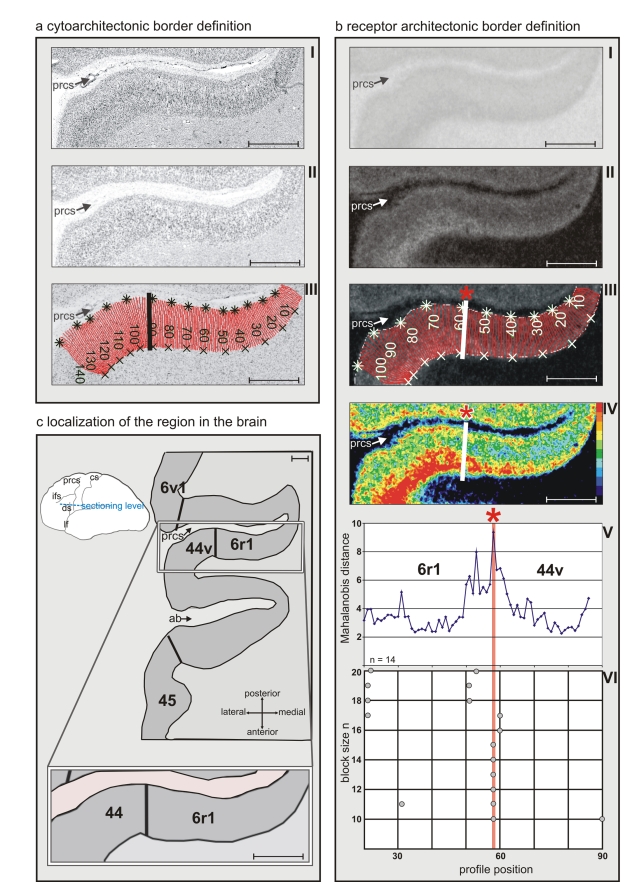
Algorithm-based detection of borders [39] in human brain sections. (a) Cytoarchitectonic border definition: cytoarchitecture (I), corresponding GLI image (II), and traverses covering the cortical ROI (III, numbered red lines). The position of the border (bold line) is superimposed onto the GLI image. GLI is an indicator of the volume fraction of cell bodies [73]. (b) Border definition in the receptor autoradiograph: receptor autoradiograph showing the distribution of glutamatergic kainate receptors (I), linearized image (II), traverses including the position of the detected border (bold line) superimposed on the linearized image (III), color-coded receptor autoradiograph (here and in the following graphs the color scale indicates to the concentration of the receptor in fmol/mg protein (IV), Mahalanobis distance function for a block size of *n* = 14 profiles (V), localization of significant peaks in the Mahalanobis distance function in dependence on the block size (*p*<0.05; VI). The Mahalanobis distance was measured between blocks of profiles (ten to 20 profiles). The border at profile number 59 (asterisk) was reproduced for different block sizes. (c) Scheme of a horizontal section through the posterior inferior-frontal human cortex including the border between areas *44v* and *6r1*. Scale bar, 5 mm. ab, ascending branch of the lateral fissure; cs, central sulcus; ifs, inferior frontal sulcus; lf, lateral fissure; prcs, precentral sulcus.

**Figure 4:**
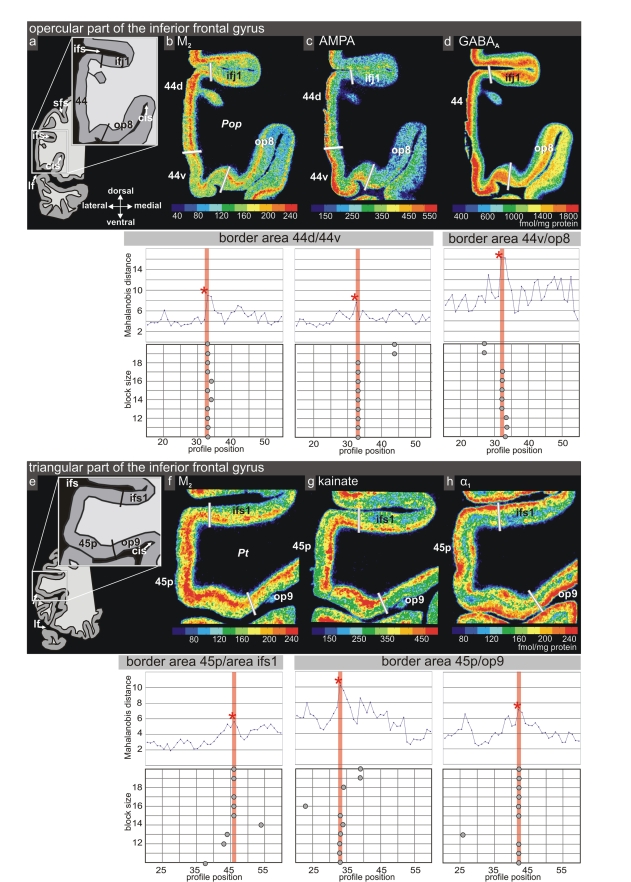
Receptor architecture the inferior frontal gyrus in coronal sections. (a, e) location of the opercular (*Pop*) and triangular parts (*Pt*) in the coronal sections. The border between areas 44 and *op8* is characterized by a decrease in receptor density of AMPA and GABA_A_ receptors mainly in the more superficial layers, and a decrease of M_2_ receptor density both in more superficial and deeper cortical layers (b–d). The receptor distribution of cholinergic M_2_, glutamatergic kainite, and noradrenergic α_1_ receptors in the triangular part is shown in (f–h). The ventral border of area 45 with area *op9* was discernible by a decrease in kainate and M_2_ receptor densities and an increase in α1 receptors. For each receptor, the Mahalanobis distance function is shown for a block size of *n* = 15 profiles together with a graph showing the dependency of the location of maxima on the block size. Red asterisk indicates the significant maximum of the Mahalanobis distance function. The areal border is indicated by a consistent occurrence of significant maxima (red frame). White dotted lines indicate the receptor architectonic subdivision of area 44 into a dorsal (d) and a ventral (v) part. Cis, circular insular sulcus; ifs, inferior frontal sulcus; sfs, superior frontal sulcus; lf, lateral fissure.

Eight architectonically defined cortical areas were identified in the posterior inferior-frontal and precentral cortex. In addition to the Brodmann areas 44, 45, 4, 6, and 47, three new areas, areas *op8* and *op9* in the frontal operculum and area *6r1* in the ventral part of the precentral sulcus (Figure 2), were found and delineated by quantitative cytoarchitectonic and receptor architectonic mapping (Figure 3). The Brodmann areas 44 and 45 could be subdivided into *44d* and *44v*, as well as *45a* and *45p*. Furthermore, five new areas adjoining our region of interest were identified, but not completely delineated in the present study: areas *6v1* and *6v2* as parts of premotor area 6, area *ifs1* located in the inferior frontal sulcus, and areas *ifj1* and *ifj2* located at the junction of the inferior frontal and the precentral sulcus (Figures 4–7).

**Figure 5 pbio-1000489-g005:**
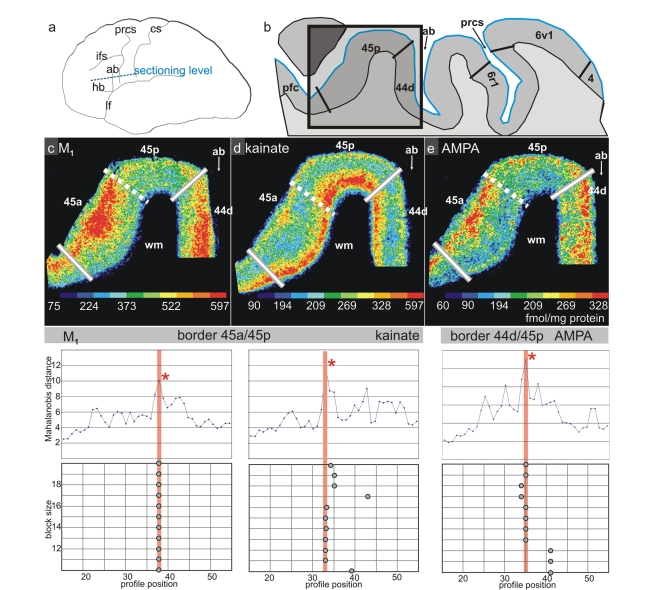
Receptor distributions of the cholinergic M_1_, and the glutamatergic kainate and AMPA receptors in a horizontal section. The border between areas 44 and 45 is characterized by a decrease in densities of M_1_ and AMPA receptors in the more superficial layers in area 45 as compared to 44, and an increase of kainate receptor density in 45. A subdivision of area 45 is indicated, dividing it into an anterior (a) and a posterior (p) part. This subdivision is indicated by differences between both areas in M_1_ and AMPA receptor densities in the more superficial layers. The graphs below show the Mahalanobis distance functions (block size of *n* = 14 profiles) together with the dependency of the location of main maxima on the block size for the border between areas *45a* and *45p* (M1 and kainate receptors), as well as for the border between areas 44 and *45p* (AMPA receptor). Designation as above. Dotted white lines indicate the receptor architectonic subdivision of area 45. ab, ascending branch of the lateral fissure; cs, central sulcus; prcs, precentral sulcus; tr, triangular part of the inferior frontal gyrus.

**Figure 6 pbio-1000489-g006:**
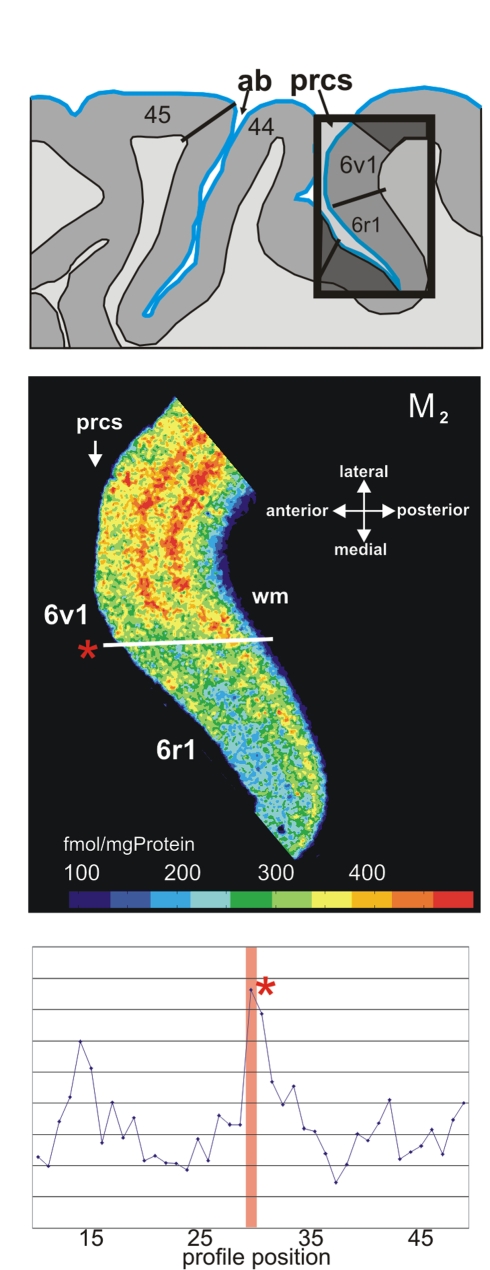
Receptor architectonic borders of area *6r1* with area 6v1 based on cholinergic M_2_-receptor distribution. Demonstration of the border (*) in a horizontal section of the posterior wall of the precentral sulcus and location of the ROI. The border between area *6v1* and area *6r1* is characterized by a decrease of M_2_ receptor density. prcs, precentral sulcus; wm, white matter. The graphs demonstrate quantification of borders. Designation as above.

**Figure 7:**
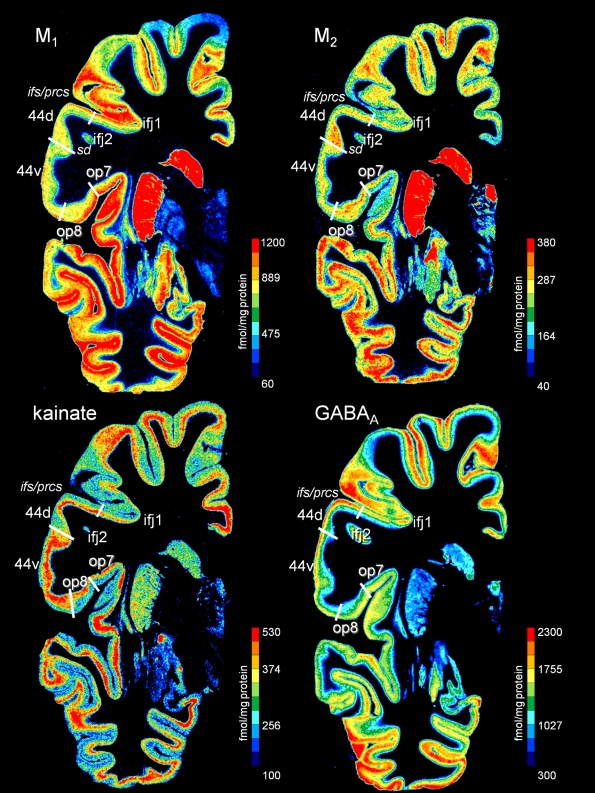
Receptor mapping in whole hemispheric human brain sections. Distribution of the cholinergic muscarinic M_1_ and M_2_ receptors, the glutamatergic kainite, and GABA-ergic GABA_A_ receptors in a neighboring coronal sections of a complete human hemisphere (level 32). The receptor concentrations are indicated in fmol/mg protein and color coded according to the color bar on the right of each section. Designation as above.

### Cytoarchitecture of Cortical Areas

Both opercular areas *op8* and *op9* were dysgranular, i.e., they showed a faint but recognizable layer IV (Figure 2). In this respect, they were similar to area 44 [26], but different from area 45, which has a well-developed inner granular layer (i.e., granular type of a cortical area). Sporadically, large pyramidal cells were found in layer III of area *op8*; they were smaller, however, than those of area 44. The columnar and laminar arrangement was less regular in area *op8* than in area 44. Compared to the dorsally adjoining area 45, *op9* showed a higher cell density, and a less regular cellular distribution. Layer III of area *op9* contained pyramidal cells that were smaller and less frequent than those in area 45.

In contrast to the neighboring, purely agranular ventral area 6, area *6r1* was almost dysgranular; it displayed a subtle layer IV (Figure 2). In comparison with rostrally adjacent, typical dysgranular area 44, layer IV of area *6r1* was even thinner and not continuous. Large pyramidal cells in deep layer III were found that were similar to those of areas 6 and 44. Similar to area 6, the laminar differentiation of area *6r1* was weak, i.e., all cortical layers from layer II to VI showed an approximately similar cell packing density.

### Receptor Architecture of Cortical Areas

Receptor architectonic borders were identified by differences in density and lamination patterns of the receptor binding sites using an observer-independent method (Figure 3; Table 2) [39].

**Table 2:** Normalized receptor densities and standard deviations for each ligand and each area (averaged over all cortical layers).

Area	AMPA	kainate	GABA_A_	M_2_	M_1_	α_1_
**4**	169±70	267±42	820±212	125±9	267±76	201±46
**6**	180±56	356±86	1002±269	163±52	331±110	259±77
***6r1***	154±60	309±66	1100±244	145±46	371±93	273±56
**44**	189±82	333±108	1250±310	176±67	355±117	250±81
**45**	196±78	336±117	1270±349	176±56	307±71	232±91
**47**	291±118	354±19	1188±244	131±32	356±29	182±63
***op8***	214±38	324±143	1254±216	178±46	335±94	307±124
***op9***	233±25	307±163	1386±369	163±64	319±78	271±136

The areal densities were averaged over all hemispheres resulting in an overall areal density value for each area and receptor. Areas 44 and 45 were not divided in *44d* and *44v*, or *45a* and *45p*, because all these areas were not present in all brains studied here.

**Figure 8:**
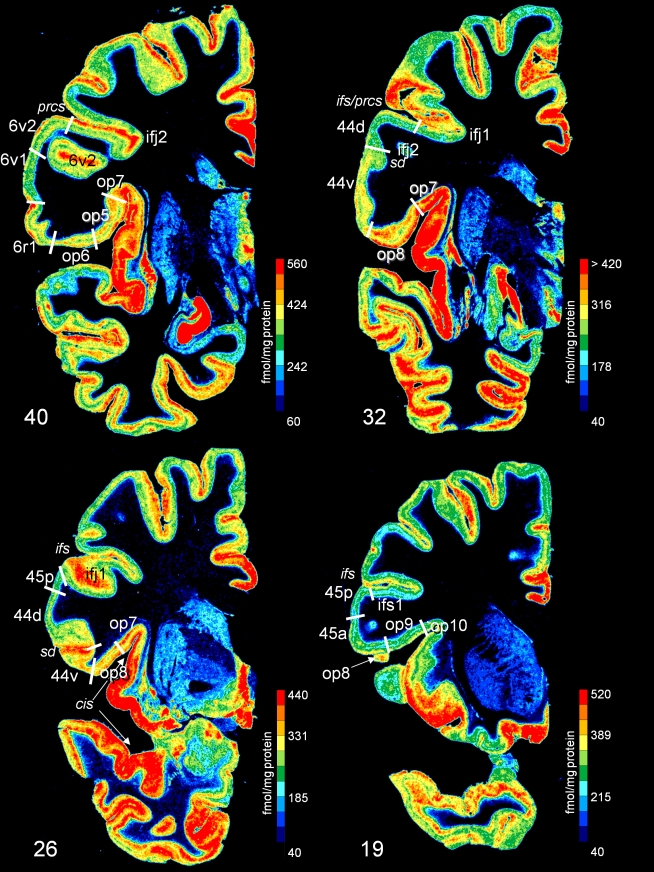
Topographical relationship of receptorarchitectonic areas in a series of four coronal sections (noradrenergic α1 receptor, from caudal to rostral at levels 40, 32, 26, and 19. The receptor concentrations are indicated in fmol/mg protein and color coded according to the color bar on the right of each section. Designation as above.

Area 44 was divided by receptorarchitectonic differences into two areas—a more dorsal *44d* and a ventral *44v*. Additionally, area *44v* appears more posterior than *44d*, and *44d* reached out to more anterior levels than *44v*. Most pronounced differences between both areas were found in muscarinic M_2_, AMPA, and α1 (Figures 4b, 4c, 7 and 8) receptors; the remaining receptors and the cytoarchitecture did not clearly separate these two areas (Figures 7 and 8).

The posterior border of area *44v* with caudally adjacent area *6r1* was particularly well delineated by kainate (Figure 3b), GABA_A_, and α_1_ receptors. The supragranular layers of area *44v* have considerably higher densities of glutamatergic AMPA and GABAergic GABA_A_ receptors compared to those of the adjacent area *op8* (Figure 4c and 4d). The borders between areas *44v* and *op8* were found at precisely the same localization in all receptor types indicative of this border (Figure 4b–4d).

The dorsally adjacent area of the inferior frontal junction region (*ifj1*) had lower receptor densities of M_2_, AMPA, and GABA_A_ receptors than area *44d* (Figure 4b–4d).

The border between area 45 and area 44 was detected by all receptors. The receptor densities revealed a subdivision of area 45 into an anterior (area *45a*) and a posterior (area *45p*) part, indicated by a lower density of M_1_ and AMPA receptors (Figure 5c and 5e) in the supragranular layers of *45p* compared to *45a*. Furthermore, the receptor density of the noradrenergic α1 receptor in *45p* was lower than in *45a* (Figure 8). The laminar distribution pattern in area 45 was similar to that of area 44 (Figure 5), but lower mean (averaged over all cortical layers) densities of the α_1_ (Figure 8), AMPA, and M_1_ (Figure 5) receptors clearly separated *45p* from *44d*. *45p* had a higher concentration of M_2_, kainate, and α_1_ receptors than the dorso-rostral neighboring area *ifs1* (Figure 4f–4h). The ventral border of area *45p* with area *op9* was indicated by higher M_2_ and kainate receptor densities (Figure 4f and 4g). *45a* had higher M_1_, kainate, AMPA (Figure 5c–5e), and α_1_ receptor densities in the supragranular layers than the rostrally adjacent prefrontal cortex.

The border between area 6r1 and area 6v1 was revealed by higher M_2_ (Figure 6) and lower α_1_ (Figure 8) receptor densities in 6r1, whereas the border of area *6r1* with *44v* was indicated by changes in kainate (Figure 3) and α_1_ receptors.

### Relationship of Cytoarchitectonic and Receptor Architectonic Parcellation

Cytoarchitectonic borders coincided with changes in the laminar distribution patterns of several or all receptor binding sites. For example, the border between areas 44 and 45 was identified in all six receptor types (AMPA, kainate, GABA_A_, M_1_, M_2_, and α_1_). However, not all borders could be demonstrated by changes in the laminar distribution patterns of all receptors; e.g., the border between areas *6r1* and 6v1 was reflected by changes in M_2_, α_1_, and kainate receptors, but less well by GABA_A_ and M_1_ receptors. The border between areas *44d* and *44v* was labeled by α1 (Figure 8) and muscarinic M_2_ (Figure 7) receptors, but less visible in the autoradiographs of kainate and GABA_A_ receptors (Figure 7).

### Topography of Cortical Areas

**Figure 9:**
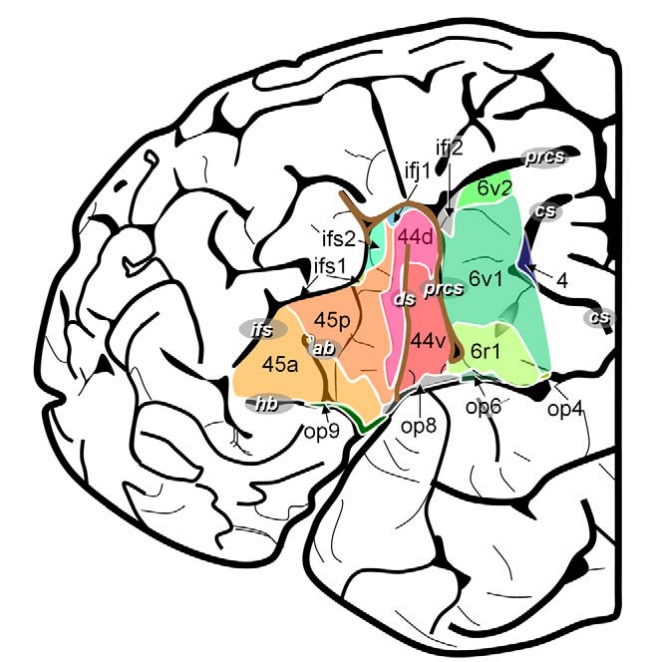
Extent of delineated areas projected to the lateral surface of an individual postmortem brain. Same hemisphere as shown in Figure 7.

The topography of areas and their spatial relationship is illustrated in a series of four coronal sections of a complete hemisphere (Figure 8). The border between ventral area 6 and caudally adjoining area 4 was located in the anterior wall of the central sulcus or the posterior portion of the precentral gyrus. Area 6 always occupied the free surface of the precentral gyrus. In some cases, it reached the lateral fissure. The receptor distribution showed a subdivision of the ventral part of area 6: two new areas, *6v1* and *6v2*, were defined in addition to area *6r1* (Figure 8). Both areas were agranular, and showed the typical cytoarchitectonic laminar pattern of area 6 as described by Brodmann [5]. They differed, however, in their receptorarchitecture, e.g., by the noradrenergic receptor (Figure 8). Dorsally to *6r1*, area *6v1* was found, which differed itself from the more dorsally adjoining premotor area *6v2* by a lower α_1_ receptor density (Figure 8, level 40). Rostrally of areas *6v1* and *6v2*, area *6r1* was located within the precentral sulcus (Figure 6). Area 44 had common caudal borders with *6r1*, *6v1*, and *6v2*. Medio-ventrally, *6r1* was adjacent to the new opercular area *op6* (Figure 8, level 40). Area *6r1* separated area *44v* from the ventral and more posterior parts of area 6 on the free surface of the brain (Figure 9).

*44v* adjoined *6r1* rostrally and covered the free surface of the opercular part of the inferior frontal gyrus. The position of the border between both areas varied in the anterior wall of the inferior precentral sulcus. The dorsal border of *44d* was found in the ventral wall of the inferior frontal sulcus; the dorsal neighbors were *ifj2* (at more caudal levels) and *ifj1* (at more rostral levels). The ventral border of *44v* with the opercular area *op8* was located at varying positions deep in the frontal operculum (levels 32 and 26 of Figure 8).

Area 45 occupied the triangular part of the inferior frontal gyrus anterior to area 44. The border between areas 44 and 45 (Figure 5) was found either within the ascending branch of the lateral fissure or on the free cortical surface of the inferior frontal gyrus, e.g., between the diagonal sulcus and the ascending branch as illustrated in Figures 8 and 9. The ventral border of *45a* with the opercular area *op9* (Figures 4f–4h and 8 at level 19) was located at varying positions at the entrance to the Sylvian fissure. Areas *op8* and *op9* were regularly found ventral to *44v* and *45a*, respectively. Area 47 occupies the orbital part of the inferior frontal gyrus; it reached only the most rostral part of area 45, and was located rostral to area *op9*. Thus, the part of area 47 at the border to area 45 was most likely area 47/12l as described by Öngür et al. [40]. Area 45 bordered dorsally to areas within the inferior frontal sulcus; an example (area *ifs1*) is shown at level 19 of Figure 8. Area *ifs1* differed in its receptor pattern from dorsally adjacent lateral prefrontal areas, and was restricted to the depths of the inferior frontal sulcus.

### Multiple Receptor Analysis of Architectonic Areas and Similarity Criteria

**Figure 10:**
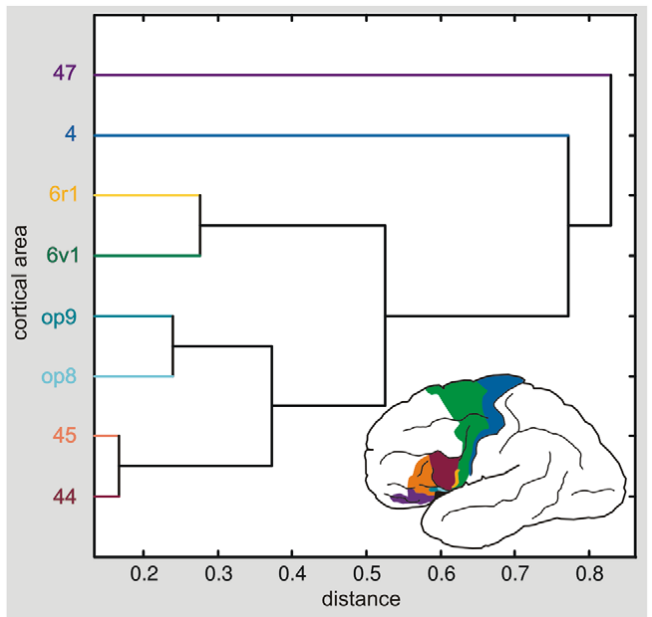
Hierarchical cluster analysis of the posterior inferior-frontal areas based on quantitative receptor architectonic data. Euclidean distances were calculated as a multivariate measure for interareal differences. A small Euclidean distance between areas, e.g., between areas 44 and 45 or areas *op8* and *op9*, indicates a high similarity in their receptor architectonic organization. The graph shows that areas 47 and 4 differ maximally from the group of areas. Areas 44 and 45 were not divided in *44d* and *44v*, or *45a* and *45p*, because all these areas were not present in all brains studied here.

Each of the areas in the inferior frontal and precentral gyri showed a distinct receptor pattern as defined by six receptor types. A canonical analysis of receptor densities in all brains and hemispheres demonstrated differences and similarities in the receptor distribution pattern, and quantified receptor architectonic differences by multivariate distances (Figure 10). The hierarchical cluster analysis showed that the prefrontal area 47 was most different from all the other areas, i.e., areas 4, 6, 44, 45, *6r1*, *op8*, and *op9* (Figure 10). On subsequent levels of the cluster tree, area 4 differed from the remaining areas. In a next step, areas 6 and *6r1* appeared in one cluster, separated from areas *op8*, *op9*, 44, and 45. On the lowest level, a distinction into two subclusters was found: one comprising areas *op8* and *op9*, and the other areas 44 and 45.

### Interhemispheric Differences

**Figure 11:**
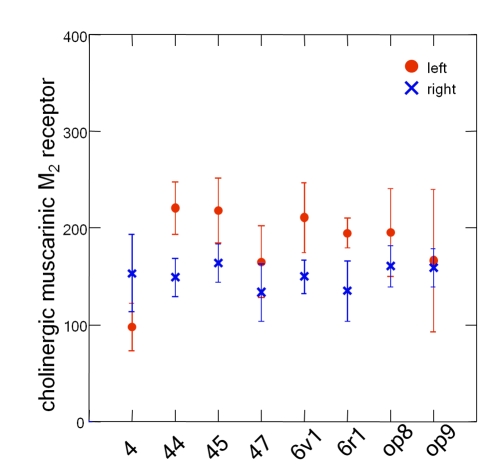
Interhemispheric differences in the concentration of receptor binding sites of the muscarinic M^2^ receptor per area. This receptor type differed significantly between left and right hemispheres with higher values on the left than on the right (*p*<0.05). Means in fmol/mg protein and standard errors of arithmetic means.

Interhemispheric differences in receptor densities were tested in three steps. First, we tested the left–right difference of all areas and receptors together using a discriminance analysis (Wilks Lambda). The densities differed significantly between the left and the right hemispheres: the overall *p*-value indicated a significant effect of hemisphere on the receptor density (*p* = 0.0091). Second, this overall interhemispheric difference (left over right) was mainly caused by the cholinergic muscarinic M_2_ receptors. It showed a left-larger-than-right asymmetry, as demonstrated by a subsequent univariate *F*-test (*p* = 0.003; Table 3). Left–right differences of each of the remaining receptors did not reach significance (*p*>0.05) if tested for each receptor type separately (Table 3). Third, if the areas were studied separately, M_2_ receptor densities of areas 44, 45, 6v1, and *6r1* were left > right, whereas area 4 showed an inverse pattern (Figure 11). Among these areas, the left–right difference for area 44 was most pronounced (*p*<0.05).

**Table 3:** Normalized receptor densities and standard deviations (averaged over all areas) for each receptor type.

Receptor Type	Left	Right	*p*-Value
**AMPA**	211.14±63.74	192.91±56.81	>0.05
**GABA_A_**	1,259.09±306.94	1,243.03±349.92	>0.05
**kainate**	326.66±112.02	367.89±81.42	>0.05
**M_2_**	199.97± 64.80	151.88±33.44	0.003
**M_1_**	439.50±281.42	350.96±99.09	>0.05
**α_1_**	273.39±104.56	274.82±123.41	>0.05

Designation as in Table 2.

## Discussion

The cerebral cortex is subdivided into structurally and functionally distinct cortical areas. Areas 44 and 45 of the anterior speech zone, Broca's region, are supposed to represent the cytoarchitectonic correlates. Homologues of these two areas have been described in nonhuman primates. Comparative studies in macaque brains provided evidence, however, that a simple subdivision of this region into two areas is not sufficient and obscures the highly differentiated organization: (i) area 45 is parcellated into an anterior and a posterior part, which differ in their connectivity [41],[42]; (ii) the transitional zone from motor cortex to Broca's region contains areas within F5, possibly involved in different aspects of motor control and cognitive functions [28],[29]. Thus, we hypothesized that Broca's region of the human brain shows a more complex segregation than assumed until now.

The present study provided a combined analysis of six transmitter receptors and cytoarchitecture in Broca's region and the frontal operculum in order to test this hypothesis. The ventral premotor cortex and neighboring prefrontal areas have also been included in order to achieve a more comprehensive view of the inferior frontal cortex and its segregation from the neighboring motor and prefrontal cortex.

The selection of the areas of the present study aimed to consider the relevant regions, and to provide an anatomical correlate of different concepts regarding the functional segregation of the inferior premotor and neighboring Broca region. Activations in the vicinity of areas 44 and 45 have been reported not only in language, but also in motor tasks [13],[19], in experiments focusing on the integration of semantic information from speech and gestures [43], and other tasks requiring cognitive control [44],[45]. For an overview about the role of motor and premotor cortices in language processing see [46]. A recent study argued that the human action observation—action execution mirror circuit—is formed by the inferior section of the precentral gyrus plus the posterior part of the inferior frontal gyrus (plus the inferior parietal lobule) [47]. As a consequence, parts of the ventral area 6 and area 44 would belong to the mirror system. The inferior frontal cortex, including Broca's region and the ventral premotor cortex, has been conceptualized as a region representing complex, systemic dependencies, regardless of modality and use: Fadiga and coauthors have speculated that this capacity evolved from motor and premotor functions associated with action execution and understanding, such as those characterizing the mirror neuron system [20]. Others proposed that the role of this region is associated with complex, hierarchical or hypersequential processing [48]. Morin and Grèzes provided arguments, on the basis of a review of 24 fMRI studies examining activations in areas 4 and 6, that the ventral precentral gyrus with area 6, and not area 44, shares the visual properties of mirror neurons found in area F5 of the macaque brain [32].

The present receptorarchitectonic study resulted in a novel parcellation of the inferior frontal cortex. Three new areas, *op8*, *op9*, and the ventral precentral transitional area *6r1*, were identified. Their borders were proven by significant changes in the laminar patterns of cyto- and receptorarchitecture using an algorithm-based method for the detection of borders [39]. Both opercular areas, *op8* and *op9*, were separated from the dorsally adjoining areas 44 and 45 by their receptor distribution pattern. Previous studies have shown that areas of similar functions show similar receptor patterns and differ from those with other properties [34]. The higher the functional similarity between two cortical areas, the more similar are their receptor distribution patterns [35]; similarities in receptor architecture between areas 44 & 45 on the one hand, and areas *op8* & *op9* on the other, suggest a corresponding functional segregation. Indeed, functional representations of hierarchically and nonhierarchically structured sentences [25] correlate with the clustering based on receptor architecture: Whereas the deep frontal operculum (where *op8* and *op9* are located) was activated during the processing of nonhierarchically and hierarchically structured sequences, areas 44 and 45 were only activated during the processing of hierarchically structured sequences that mimicked the structure of syntactically complex sentences in natural languages [25]. A diffusion-weighted magnetic resonance imaging study revealed a separation of Brodmann area 44, 45, and the deep frontal operculum on the basis of differences in their connectivity [49].

The analysis of the receptor distribution patterns using hierarchical clustering supports the notion that areas 44 and 45 are closely related. It disagrees with those concepts, which attributed Broca's region solely to either area 44 [50] or area 45 [51], or to a cortical assembly combining areas 44 and 45 with area 47 [52].

Area 47 was most distinct from any of the analyzed areas as shown in the cluster analysis, thus suggesting a different functional involvement. The present data, therefore, imply that it is not meaningful to attribute activation clusters obtained in functional imaging studies to a region labeled as “45/47,” since these are two independent, structurally and functionally, completely different cortical areas.

The newly described area *6r1* showed cyto- and receptorarchitectonic features that places it in between area 44 and area 6. The area was called *6r1* in order to underline that it is located rostrally from premotor area 6; “1” indicates that this is the first area of a group of areas that we expect to be located rostrally to the precentral area 6; this belt of areas is located at the transition of the motor domain to the prefrontal cortex. Because of the higher microstructural similarity of area *6r1* with the classically described Brodmann area 6 than to 44, it was labeled as “*6r1.*” When analyzing the neighborhood of area *6r1* it became obvious, that the ventral part of area 6 consists of several areas, not yet described in the human brain. At least two more areas, *6v1* and *6v2*, have been identified in the present study on the basis of receptor and cytoarchitectonic criteria. This finding supports data of a recent study analyzing the connectivity of the premotor cortex in the human brain [53]. Studies of the macaque brain already resulted in detailed parcellation schemes (for an overview of parcellation schemes see figure 1 in Belmalih et al. [28]). However, the topography and the sulcal pattern of the ventral frontal cortex differ considerably between macaque and human brains.

There are, on the other hand, also similarities of the present parcellation of the inferior frontal cortex with a parcellation found in a recent study in macaque monkeys [28]. The authors described an area F5a in the inferior arcuate sulcus bordering area 44. F5a may correspond to area *6r1* not only by its location but also by its cytoarchitectonic features. Even though area F5a is part of the agranular frontal cortex, it shows transitional features displaying granular cells as well as a relatively prominent layer V [28]. Further cytoarchitectonic studies will be necessary to compare the subdivisions of macaque F5 with human *6r1* in detail. If the abilities associated with Broca's region have evolved from premotor functions [54], area *6r1* may be interpreted as some kind of “transitional” area between the motor cortex and Broca's region. The identification of area *6r1* implies that area 44 does not border the ventral premotor area 6 over its full extent as supposed by other maps [5],[41]. Future cytoarchitectonic mapping studies would help to understand the extent of the inferior frontal lobe areas and its intersubject variability.

New areas were also found in dorsa-caudally adjacent areas of area 44. Two areas, *ifj1* and *ifj2*, were distinguished (Figure 7), which are located immediately rostrally to premotor area 6. Both were found at the junction of the inferior frontal and the precentral sulcus, and, therefore correspond to the previously described inferior frontal junction region [55]–[57]. In contrast to earlier observations, however, here we identified two new areas instead of one, which had been hypothesized on the basis of functional imaging experiments, for example during task switching [56],[58]. The functional difference between *ifj1* and *ifj2* remains to be further elucidated.

Additional new neighboring areas (e.g., *ifs1*) were located in the depths of the inferior frontal sulcus where, according to Brodmann's map, areas 46 or 9 would be expected (Figures 4 and 7b at level 19). The present analysis of the complete coronal sections demonstrates that a series of small areas occupies the sulcus. These areas in the inferior frontal sulcus are different by their receptorarchitecture from the dorsally adjacent areas of the dorso-lateral prefrontal cortex, and, therefore, have not been labeled as areas 46 and 9, but *ifs1*, etc. The analysis and mapping of these new areas, again, represents an independent research project, which would exceed the present study.

We provided evidence for a further parcellation within area 44 and area 45. Differences in the laminar receptor distribution patterns of AMPA and M_1_ receptors argue for a subdivision of area 44 into a ventral and dorsal part extending earlier cytoarchitectonic findings [26]. A dorso-ventral subdivision of area 44 is a putative correlate of functional differentiation within this area as indicated by recent imaging studies: Molnar-Szakacs et al. [59] reported activations in the dorsal part of area 44 during observation and imitation of actions, whereas the ventral part was activated during imitation, but not during observation of actions. The ventral, but not the dorsal part, was activated during the imagery of movement [14]. Finally, an activation in the ventral part of area 44 was found for syntactic processing during language production [10] and comprehension [25], whereas the dorsal opercular part (where 44 is found) was involved in phonological processing [9].

The laminar receptor distribution patterns subdivided area 45 into an anterior and a posterior part on the basis of differences in the density of noradrenergic α1 M_1_, AMPA GABA_A_ receptors. The subdivision of area 45 agrees with a recent study comparing the cytoarchitectonic organization in the human and macaque cortex [60]: Petrides and Pandya divided area 45 into a more anterior part (area 45 A) and a more posterior part (area 45 B, located anterior to area 44) using the width of layer II as the distinguishing feature (being narrower in area 45 A than in 45 B). This finding was further supported by demonstrating differences in connectivity [41].

The outcome of the present study is a considerably detailed parcellation of Broca's region and the immediately surrounding cortex. Some of the new units described here can be assigned to regions covered by Brodmann areas and defined by his nomenclatural system [5]. In such cases, we keep Brodmann's numbering system and define the new units by Brodmann's number and an additional letter and/or number (e.g., *6r1*, *44a*, *44p*). In other cases, new cortical units could not be reliably assigned to a Brodmann area, e.g., *op8* and *op9*. Since our new parcellation is based on an observer-independent approach and statistical tests of the significance of regional differences, we will call all cortical units “areas.”

The question, however, of how a cortical unit is defined as “area,” and what makes it special as compared to a unit called “subarea,” or an intra-areal specialization, remains. Examples of intra-areal specializations would be somatotopies in sensory and motor areas and ocular dominance columns, i.e., structures that are regionally specific to a certain degree, but subserve a common function. Currently, the concept of a “subarea” is vaguely defined, and is used inconsistently in the literature. Therefore, we adopt the term “area” throughout the article.

A central question to any study devoted to Broca's region is that of lateralization. Several studies have provided evidence that cytoarchitecture [26],[50],[61]–[64], fiber tracts [65], and macroscopical anatomy of this region are asymmetric [66]–[68]. For overviews see [69] and [70]. These structural asymmetries were interpreted as putative correlates of functional lateralization. The present study revealed significant interhemispheric differences in the receptor concentrations when all six receptor types were taken together. A subsequent analysis was performed in order to identify the receptor type that contributed most to this finding. The cholinergic M_2_-receptor showed the only significant left–right difference. Interhemispheric differences of receptors in Broca's region have not been reported up to now.

In conclusion, the novel parcellation of the ventro-lateral frontal cortex and Broca's region provides a new anatomical basis both for the interpretation of functional imaging studies of language and motor tasks as well as for homologies between human and macaque brains. It will, therefore, contribute to the understanding of the evolution of language. The analysis of the receptor distribution sheds new light on the organizational principles of this region. This direction is a further step from a rigid and exclusively cytoarchitectonic parcellation scheme as introduced by Brodmann 100 years ago [71] towards a multimodal and functionally relevant model of Broca's region and surrounding cortex.

## Materials and Methods

### Processing of the Post Mortem Brains

Adult post mortem brains of body donors were removed from the skull within less than 24 h post mortem in accordance with legal requirements (Table 1). None of the subjects had clinical records of neurological or psychiatric disorders. Six hemispheres were dissected into coronal slabs of approximately 30 mm thickness (Figure S1). Tissue blocks containing the posterior part of the inferior-frontal cortex were dissected from six hemispheres of three brains and sectioned horizontally. The tissue was frozen and stored at −70°C. Serial sections (thickness 20 µm) were prepared at −20°C using a large-scale cryostat microtome. The sections were thaw mounted onto glass slides (Figure S1).

### Tissue Processing for Quantitative Receptor Autoradiography

The following receptor binding sites were studied: glutamatergic AMPA and kainate receptors, GABAergic GABA_A_ receptors, cholinergic muscarinic M_1_ and M_2_ receptors, and noradrenergic α_1_ receptors (Table S1). Alternating brain sections were incubated with the receptor-specific tritiated ligands only, the tritiated ligands, and respective nonradioactive compounds (for measurement of nonspecific binding), or were stained for the visualization of cell bodies [72]. Thus, a group of serial sections at the same sectioning level demonstrates the different receptor types, and the regional cytoarchitecture (Table S1; for details see Zilles et al. [35]). Since nonspecific binding was less than 10% of the total binding in all cases and receptor types, the total binding was accepted as an estimate of the specific binding. The labeled sections were coexposed with plastic standards of known concentrations of radioactivity (Amersham) to β-sensitive films. The films were developed after 10–12 wk of exposure depending on the receptor type, and digitized using the KS400 image analyzing system (Zeiss). The grey value distribution in the autoradiographs is nonlinearly correlated [35] with the local concentrations of radioactivity (Figure S1), which represent the regional and laminar distribution of receptor binding sites. Therefore, the known concentration of radioactivity of the coexposed standards (Figure S1d, bottom right) enables the nonlinear transformation of grey values into receptor binding site concentrations in fmol/mg protein (linearized images). For improved visualization of the regionally different receptor concentrations, the linearized images were contrast enhanced, smoothed, and pseudo-color coded in a spectral sequence (Figure S1f).

### Quantitative Architectonic Analysis

Neighboring sections were stained for cell bodies to demonstrate the cytoarchitecture. Rectangular regions of interest (ROIs) containing area 44 and 45 of Broca's region and neighboring areas were defined. Images (1,376×1,036 pixels; spatial resolution 1.02 µm per pixel) of the ROIs were acquired using a microscope equipped with a digital camera (Axiocam MRm, Zeiss) and a scanning stage. A high-resolution image of the total ROI was then assembled from the individual tiles employing the KS 400 system (Zeiss; Figure 3a I). Grey level index (GLI) images of the ROIs were calculated by adaptive thresholding with a spatial resolution of 16×16 µm. The resulting GLI image (Figure 3a II) represents in each pixel the local volume fraction of cell bodies [73].

Borders between cortical areas were identified in the receptor autoradiographs as well as in the cell body–stained sections using an algorithm-based approach and multivariate statistical analysis [39]. Therefore, laminar profiles of the GLI distribution were extracted in the cell body–stained sections using MATLAB-based software (MATLAB 7.2) (Figure 3a III). Laminar profiles were also obtained for the binding site densities in the autoradiographs (Figure 3b III). A feature vector was calculated for each profile, which described the shape of each profile, i.e., the cyto- or receptorarchitecture [39]. Differences in the shape of the profiles were quantified by a multivariate distance measure, the Mahalanobis distance. A subsequent Hotelling's *T* ^2^ test with Bonferroni correction for multiple comparisons was applied for testing the significance of the distance. Profiles sampled from one and the same cortical area were similar in shape, resulting in small Mahalanobis distances. Profiles sampled from different sides of a cortical border differed in shape and resulted in large distances.

To improve the signal-to-noise ratio, distances were calculated not between single profiles, but blocks of ten to 20 adjacent profiles. The position of a significant maximum in the Mahalanobis function was interpreted as a cortical border, if it was found for different block sizes (Figure 3b VI), and if it was reproduced in a similar position in adjacent sections. These criteria allowed the rejection of borders caused by artifacts due to tissue processing, or blood vessels.

### Hierarchical Cluster Analysis

For each receptor, the density averaged over all layers of a cortical area was calculated in a set of sections/autoradiographs of each hemisphere separately. These mean receptor densities were averaged over all hemispheres resulting in a mean areal density value for each area and receptor type.

The density values of all six receptors studied were combined into a receptor feature vector for each area. A hierarchical cluster analysis (MATLAB 7.2) was performed in order to analyze receptor architectonic similarities and dissimilarities between the different areas (Euclidean distance, Ward linking). The higher the similarity between two cortical areas, the smaller was the Euclidean distance between their feature vectors.

### Interhemispheric Differences

A one-way ANOVA analysis (Systat 12) was performed to test for interhemispheric differences in receptor densities of all areas and receptors together. The factor “hemisphere” had two levels: left and right. Cases with missing values were excluded from the analysis. A post hoc univariate *F* test was performed in order to identify receptor types that contributed mostly to overall interhemispheric differences. Finally, we tested interhemispheric differences for each cortical area and receptor. The *p*-level was set to 0.05.

## Supporting Information

Figure S1 **Preparation of receptor autoradiographs.** (a) Lateral view of a left hemisphere showing the gross anatomy of the posterior inferior-frontal cortex and the sectioning level (dotted blue line). (b) Sectioning of slabs of brain tissue on the cryostat-microtome (20 µm thickness). (c) Sections are spread onto frozen slides (−20°C) and thaw mounted onto the slides. (d) After incubation with 3H-labeled ligands the sections are exposed to β-radiation-sensitive film. The developed films show the local concentrations of radioactivity as spatial distribution patterns of grey values. Standards with known concentration of radioactivity are coexposed (bottom right) together with the sections. (e) The concentrations of radioactivity of the standards are used to establish nonlinear transformation curves that convert the grey values into linearly spaced concentrations of radioactivity in fmol/mg of protein (linearized image). (f) Receptor autoradiographs are pseudo color coded to improve the visualization of regional and laminar receptor distributions. The range of receptor density is divided into 11 equal intervals, each represented by a color ranging from black to red (scale bar on the left). ab, ascending branch of the lateral fissure; cs, central sulcus; hb, horizontal branch of the lateral fissure; ifs, inferior frontal sulcus; lf, lateral fissure; prcs, precentral sulcus.
(0.27 MB DOC)

Table S1 **Binding protocols.** Six different receptor binding sites were used in this study, covering several classical neurotransmitter systems: glutamatergic AMPA and kainate; GABAergic GABA_A_; cholinergic muscarinic M_1_ and M_2_; noradrenergic α_1_. Sections were incubated with the tritiated ligand (total binding) or with the tritiated ligand plus an unlabeled specific displacer (nonspecific binding). The specific binding equals the difference between total and nonspecific binding. Since the nonspecific binding was less than 10% of the total binding in all cases and receptor types, the total binding was accepted as a good estimate of the specific binding [35].
(0.05 MB DOC)

## Abbreviations

*6r1*: ventral precentral transitional area located between area 6 and area 44 of Brodmann
*6v1*: *6v2*, ventrally located areas within a region defined as area 6 by Brodmann
GLI: grey level index
*op 5–7*: opercular areas, located caudally and ventrally to the region of interest
*op 8* and *op 9*: opercular areas 8 and 9
ROI: region of interest
