# Supplementary material for: Broca's Region: Novel Organizational Principles and Multiple Receptor Mapping
Source: PLoS Biol. 2010 Sep 21;8(9):e1000489. doi: 10.1371/journal.pbio.1000489 (PMC2943440; doi:10.1371/journal.pbio.1000489)
Supplement: Figure S1 — Preparation of receptor autoradiographs. (a) Lateral view of a left hemisphere showing the gross anatomy of the posterior inferior-frontal cortex and the sectioning level (dotted blue line). (b) Sectioning of slabs of brain tissue on the cryostat-microtome (20 µm thickness). (c) Sections are spread onto frozen slides (−20°C) and thaw mounted onto the slides. (d) After incubation with 3H-labeled ligands the sections are exposed to β-radiation-sensitive film. The developed films show the local concentrations of radioactivity as spatial distribution patterns of grey values. Standards with known concentration of radioactivity are coexposed (bottom right) together with the sections. (e) The concentrations of radioactivity of the standards are used to establish nonlinear transformation curves that convert the grey values into linearly spaced concentrations of radioactivity in fmol/mg of protein (linearized image). (f) Receptor autoradiographs are pseudo color coded to improve the visualization of regional and laminar receptor distributions. The range of receptor density is divided into 11 equal intervals, each represented by a color ranging from black to red (scale bar on the left). ab, ascending branch of the lateral fissure; cs, central sulcus; hb, horizontal branch of the lateral fissure; ifs, inferior frontal sulcus; lf, lateral fissure; prcs, precentral sulcus. (0.27 MB DOC) [file pbio.1000489.s001.pdf]

Supplement

Figure 1

Preparation of receptor autoradiographs

(a) Lateral view of a left hemisphere showing the gross anatomy of the posterior inferior-frontal cortex and the sectioning level (dotted blue line).

(b) Sectioning of slabs of brain tissue on the cryostat-microtome (20 µm thickness).

(c) Sections are spread onto frozen slides (-20° C) and thaw-mounted onto the slides.

(d) After incubation with 3H-labeled ligands the sections are exposed to β-radiation-sensitive film. The developed films show the local concentrations of radioactivity as spatial distribution patterns of grey values. Standards with known concentration of radioactivity are co-exposed (bottom right) together with the sections.

(e) The concentrations of radioactivity of the standards are used to establish nonlinear transformation curves which convert the grey values into linearly spaced concentrations of radioactivity in fmol/mg of protein (linearized image).

(f) Receptor autoradiographs are pseudo color-coded to improve the visualization of regional and laminar receptor distributions. The range of receptor density is divided into 11 equal intervals, each represented by a color ranging from black to red (scale bar on the left).

ab: ascending branch of the lateral fissure, cs: central sulcus, hb: horizontal branch of the lateral fissure, ifs: inferior frontal sulcus, lf: lateral fissure, prcs: precentral sulcus.

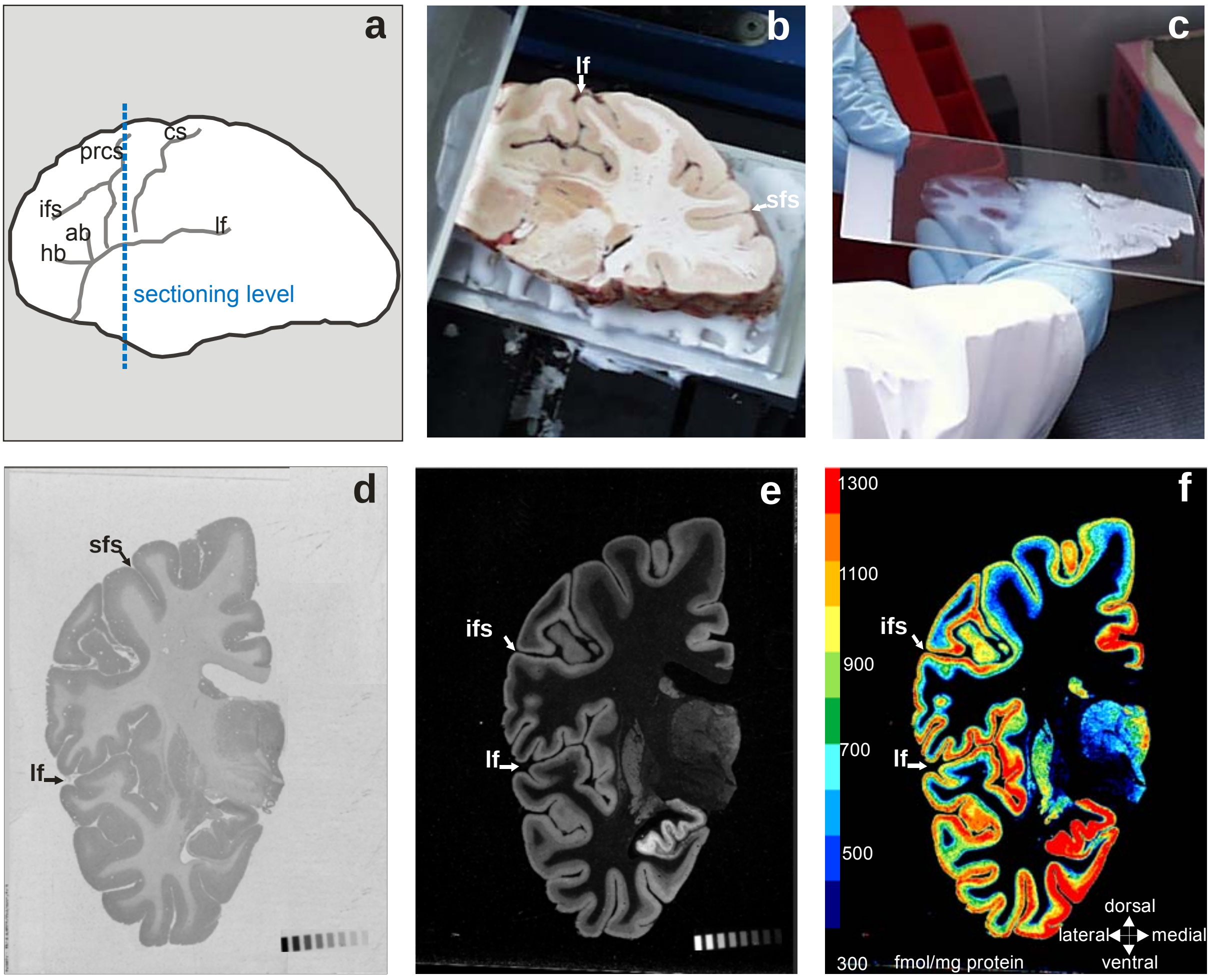

a
cs
prcs
ifs
ab
hb
lf
sectioning level
b
lf
sfs
c
d
sfs
lf
e
ifs
lf
f
1300
1100
900
700
500
300
ifs
lf
dorsal
lateral
medial
ventral
fmol/mg protein
