# Supplementary material for: Broca's Region: Novel Organizational Principles and Multiple Receptor Mapping
Source: PLoS Biol. 2010 Sep 21;8(9):e1000489. doi: 10.1371/journal.pbio.1000489 (PMC2943440; doi:10.1371/journal.pbio.1000489)
Supplement: Table S1 — Binding protocols. Six different receptor binding sites were used in this study, covering several classical neurotransmitter systems: glutamatergic AMPA and kainate; GABAergic GABAA; cholinergic muscarinic M1 and M2; noradrenergic α1. Sections were incubated with the tritiated ligand (total binding) or with the tritiated ligand plus an unlabeled specific displacer (nonspecific binding). The specific binding equals the difference between total and nonspecific binding. Since the nonspecific binding was less than 10% of the total binding in all cases and receptor types, the total binding was accepted as a good estimate of the specific binding [35]. (0.05 MB DOC) [file pbio.1000489.s002.pdf]

Supplement table 1: Binding protocols

Six different receptor binding sites were used in this study, covering several classical neurotransmitter systems: glutamatergic: AMPA, kainate; GABAergic: $GABA_A$; cholinergic: muscarinic M_1 and M_2; noradrenergic: α_1. Sections were incubated with the tritiated ligand (total binding) or with the tritiated ligand plus an unlabeled specific displacer (non-specific binding). The specific binding equals the difference between total and non-specific binding. Since the non-specific binding was less than 10% of the total binding in all cases and receptor types, the total binding was accepted as a good estimate of the specific binding (Zilles et al., 2002)

**Receptor**	**^{3}H-Ligand** [nM]	**Displacer** [µM]	**Incubation buffer**	**Preincubation**	**Main incubation**	**Rinsing**
glutamatergic	AMPA [10]	Quisqualat [10]	50 mM Trisacetat (ph 7,2) + 100 mM KSCN	3 x 10 min at 4°C	45 min at 4°C	3 x 4 s in buffer at 4°C 2 x 2 s in Aceton/Glutataldehyd at 4°C (100ml/2,5ml)
glutamatergic	Kainat [9,4]	Sym 2081 [100]	50mM Tris-citrat (pH 7,1) + 10mM Ca-acetat	3 x 10 min at 4°C	45 min at 4°C	3 x 4 s in buffer at 4°C 2 x 2 s in Aceton/Glutataldehyd at 4°C (100ml/2,5ml)
GABAergic	Muscimol [7,7]	GABA [10]	50 mM Tris-citrat (pH 7,0)	3 x 5 min at 4°C	40 min at 4°C	3 x in 3 cuvette buffer
cholinergic muscarinic M1	Pirenzepin [1,0]	Pirenzepin [10]	mod. Krebs-Ringer (pH 7,4)	20 min at 22°C fixation 20 min Bouin-steam rinsing 3 x 3 min in buffer	60 min at 22°C	2 x 5 min in buffer 3 x in Aqua bidest at 4°C
cholinergic muscarinic M2	Oxotremorin-M [0,8]	Carbachol [1]	20 mM Hepes-Tris (ph 7,5) + 10 mM $MgCl_2$	20 min at 22°C	60 min at 22°C	2 x 2 min in buffer at 4°C 3 x in Aqua bidest
noradrenergic α_1	Prazosin [0,4]	Phentolamin-mesylat [10]	50 mM Tris-HCL (pH 7,4)	30 min at 30°C	45 min at 30°C	2 x 5 min in buffer at 4°C 3 x in Aqua bidest

1. Zilles, K., Palomero-Gallagher, N., Grefkes, C., Scheperjans, F., Boy, C., Amunts, K., & Schleicher, A. (2002) *Eur. Neuropsychopharmacol.* **12**, 587-599.
